# Personal Recovery and Its Determinants Among People Living With Schizophrenia in China

**DOI:** 10.3389/fpsyt.2020.602524

**Published:** 2020-12-11

**Authors:** Yu Yu, Xi Xiao, Min Yang, Xiao-ping Ge, Tong-xin Li, Gui Cao, Ying-jun Liao

**Affiliations:** ^1^Department of Social Medicine and Health Management, Xiangya School of Public Health, Central South University, Changsha, China; ^2^Division of Prevention and Community Research, Department of Psychiatry, Yale School of Medicine, New Haven, CT, United States; ^3^Department of Psychiatry, Changsha Psychiatric Hospital, Changsha, China; ^4^Department of Geriatrics, Changsha Psychiatric Hospital, Changsha, China; ^5^Department of Health Insurance and Long Term Care, Chinese Academy of Labor and Social Security, Beijing, China; ^6^Department of Nephrology, Hunan Key Laboratory of Kidney Disease and Blood Purification, The Second Xiangya Hospital, Central South University, Changsha, China

**Keywords:** recovery, schizophrenia, predictors, disability, functioning, China

## Abstract

**Objective:** The past few decades have seen an evolution in the understanding of recovery from a clinical-based view that focuses on symptoms and functioning to a more consumer-oriented perspective that focuses on personal recovery. The present study aimed to assess personal recovery among people living with schizophrenia and determine its predictors.

**Methods:** This cross-sectional study recruited a random sample of 400 people living with schizophrenia (PLS) from twelve community health centers of Hunan, China. Recovery was assessed using the short-form 8-item Recovery Assessment Scale (RAS-8). PLS disability and functioning were assessed using the 12-item World Health Organization Disability Assessment Schedule 2.0 (WHODAS 2.0) and the Global Assessment of Functioning (GAF), respectively.

**Results:** Participants had a mean personal recovery score of 20.29 (SD: 9.31, Range: 8–40). Personal recovery was predicted by both socio-demographic and clinical characteristics. Older age (*r* = −0.17, *p* < 0.001), being female (*r* = −2.29, *p* = 0.019), and higher disability (*r* = −0.22, *p* < 0.001) were independently associated with worse personal recovery, while having a college education (*r* = 5.49, *p* = 0.002), and higher functioning (*r* = 0.09, *p* = 0.017) were independently associated with better personal recovery.

**Conclusion:** Interventions to improve recovery among PLS may be best served by reducing the impact of disability and improving functioning, with targeted interventions for individuals who are older, female and less educated in order to increase their likelihood of recovery.

## Introduction

Schizophrenia is a chronic, debilitating, severe mental illness identified by positive and negative symptoms and social and occupational dysfunction, causing huge disease burden worldwide (1, 2). Schizophrenia affects ~1% of the population and has been listed among the top 25 leading causes of disability and the top 11 leading cause of reduced years lived with disability (3, 4). Traditionally, schizophrenia has been pessimistically viewed as a disease from which there is a poor recovery, with only 20% returning to premorbid levels of functioning, 20% have continuing decline, and the rest 60% neither improve nor decline, and thus most people living with schizophrenia (PLS) need long-term medication and guardianship (5, 6). However, this pessimistic view has been challenged by abundant long-term studies demonstrating good outcomes among PLS. For instance, some long-term studies suggest that as many as 50% PLS have good outcomes and can lead a productive and satisfying life even with the illness (7, 8). Consistent with this research, there is also a growing consumer movement with a central focus on the concept of recovery (6).

The understanding of recovery has evolved in the past few decades (9–11). Historically, recovery has been defined from a clinical point of view as elimination or reduction of symptoms, functional rehabilitation (e.g., cognitive, social, and vocational) and reduced use of medical health services (12). In simple words, the term recovery indicates the absence of disease, or cure (13). This clinical-based definition has been increasingly criticized for not being suited to a prolonged and persistent disorder such as schizophrenia (6). A consumer-oriented perspective complements the clinical perspective by introducing the concept of personal recovery. Personal recovery refers to an ongoing personal process of adaptation and development to overcome the negative personal and social consequences of mental disorder (14). It includes reclaiming autonomy, developing a positive sense of the self, achieving self-determination and regaining a self-determined and meaningful life (10, 15). By this definition, PLS can still lead a personally meaningful and contributory life and strive to achieve their full potential beyond the limitations imposed by the illness itself (16, 17). Key components of personal recovery include hope, optimism, self-worth, and empowerment, which have been shown to be fundamental to well-being among PLS (18). Key processes of personal recovery include connectedness, hope, identity, meaning, and empowerment, referred to as CHIME framework, which has been the guiding theoretical framework for the conceptualization of personal recovery (19).

Ever since the conceptualization of personal recovery, evidence has shown people living with schizophrenia enjoy personal recovery despite impaired clinical recovery (20, 21). A recent meta-analysis on the association between personal recovery and clinical recovery also showed only small to medium association between them, indicating personal recovery and clinical recovery are two distinct concepts, yet complements each other (22). As a multi-domain concept, personal recovery has been operationalized in many different ways, with various scales developed to measure personal recovery. A systematic review of these measures has identified the Recovery Assessment Scale (RAS) as the most widely used scale worldwide, while the Process of Recovery (QPR) most closely maps to the CHIME framework of recovery (23).

The articulation of a conceptualization of recovery among PLS has sparked excitement in studying factors that influence recovery to guide health interventions. Past studies have identified a series of factors that predict recovery, which may generally be categorized into two types of factors: socio-demographic and clinical. Some common socio-demographic predictors of better recovery include: being female, older age at onset, higher education, full employment, and not living alone (24–26). For clinical predictors, both cross-sectional and longitudinal studies have identified various factors that predict better recovery, such as shorter duration of untreated psychosis, better social functioning, better premorbid adjustment, fewer negative symptoms at baseline, no substance abuse at baseline, and adherence to medication (25–29). However, most studies focused on the predictors of clinical recovery, while few studies have investigated predictors of personal recovery. Some studies even didn't distinguish between clinical and personal recovery and combine them together to study predictors. It is thus important to study predictors of personal recovery alone. Besides, the above-mentioned predictors have been mainly reported in developed countries in western culture. Little is known about how socio-demographic and clinical factors affect personal recovery in developing countries like China.

The current study was conducted to understand recovery and its correlates among a Chinese urban community sample of PLS. Specifically, we examined both socio-demographic and clinical factors that predict personal recovery. These were intended to identify factors to consider for future intervention programs to promote recovery among PLS.

## Methods

### Participants and Procedures

This was a cross-sectional study conducted in 12 community health centers in Changsha city of Hunan Province, China from May 2019 to September 2019. All participants were recruited from China's largest demonstration project in mental health services — the “686 Program.” The “686 Program” is aimed at integrating hospital and community services for serious mental illness, with a series of services provided including a monthly free medicine distribution to registered patients (30, 31). Sample size was calculated according to the form for cross-sectional study: *n* = z^2^ P (1-P)/E^2^, where P (the prevalence of recovery) was estimated at 50% based on past studies, Z was set as 1.96 at a confidence interval of 95%, allowable error was set as 5%, the final sample size came to 384. In order to get a sample that is as representative of the Changsha City as possible, a two-stage cluster-sampling method was adopted to identify subjects. In the first stage, all five administrative districts of Changsha City were included as our sampling frame. In the second stage, 12 community health centers from the five districts were randomly selected as our final sampling frame. In each community center, we randomly selected participants based on the following inclusion criterial: adults over 18 years of age with a diagnosis of schizophrenia by the Chinese Classification of Mental Disorders-3(CCMD-3) or the International Classification of Diseases-10 (ICD-10), living with at least one family member, and able to read and communicate. Those who were younger than 18, with a diagnosis other than schizophrenia, living alone, illiterate, or having serious cognitive dysfunction thus not able to read or communicate as assessed by the psychiatrists were excluded from the study.

The study was approved by the Ethical Review Board of the Xiangya School of Public Health of Central South University (No.: XYGW-2019-029). During the monthly free medicine distribution day, a research team of 3 psychiatrists went to each health center, where all registered patients with mental illness receive their free medicine refill. A poster with detailed information about the study was posted in each health center to promote study participation. Participation included a clinical assessment on functioning by Global Assessment of Functioning (GAF), as well as completing a brief survey including socio-demographics, self-rated disability by World Health Organization Disability Assessment Schedule 2.0 (WHODAS 2.0) and personal recovery by an 8-item Recovery Assessment Scale (RAS-8). Both the clinical assessment and brief survey were implemented by the research team of 3 psychiatrists who have known the patients for a long time. The research team has received a 2-day uniform standard training on assessment prior to the formal study and tested with high inter-rater reliability. All participants were explained the study and provided written informed consent. All participants were reimbursed with RMB ¥ 10 (USD $1.4) for their participation. We used the STROBE cross sectional checklist when writing our report (32).

### Measures

#### Disability

Disability for PLS was self-assessed by the 12-item World Health Organization Disability Assessment Schedule 2.0 (WHODAS 2.0) (33) to measure disability and functional impairment. This measure covers six domains of functioning: cognition, mobility, self-care, getting along with people, life activities, and participation in society (33). Each item is rated on a 5-point Likert scale from 0- “no difficulty” to 4- “extreme difficulty” to assess the level of difficulty experienced while performing the activities. The total score ranges from 0 to 48, with higher score representing higher level of disability (34). The WHODAS 2.0 has been widely used in China with good psychometric performance established (35, 36). In the current study, the WHODAS 2.0 showed good internal consistency, with a Cronbach's alpha of 0.89.

#### Functioning

Functioning of PLS was clinician-assessed by the Global Assessment of Functioning (GAF) to measure a person's psychological, social, and occupational functioning on a hypothetical continuum of mental health-illness ranging from 1 to 100 (37). It is a one-item question, with higher score indicating better patient functioning. Examples are given for each ten-level interval. GAF has also been widely used in clinical assessment with satisfactory psychometric properties established (38, 39). In the current study we assessed the functional level of PLS over the past 1 month.

#### Recovery

The recovery of PLS was self-assessed using an 8-item Recovery Assessment Scale (RAS-8), which is a short form with two dimensions (goal and success orientation and no domination by symptoms) extracted from the most widely used standard five-dimensional 24-item RAS version (40). The domain of goal and success orientation maps onto the domains of hope and meaning from the CHIME framework and includes item 1 “I have a desire to succeed” (hope); item 2 “I have my own plan for how to stay or become well” (hope); item 3 “I have goals in life that I want to reach” (hope); item 4 “I believe that I can meet my current personal goals” (hope); and item 5 “I have a purpose in life” (meaning). The domain of no domination by symptoms loads onto the domain of empowerment from the CHIIME framework and includes item 15 “Coping with my mental illness is no longer the main focus of my life,” item 16 “My symptoms interfere less and less with my life,” and item 17 “My symptoms seem to be a problem for shorter periods of time each time they occur.” Each item is rated on a 5-point Likert scale from 1—“strongly disagree” to 5- “strongly agree.” The total score ranges from 0 to 40, with higher score indicating a better perception of recovery. In the current study, the RAS-8 showed good internal consistency, with a Cronbach's alpha of 0.91.

### Statistical Analysis

The default list wise deletion was used to deal with missing values. Scales and indices were tested for reliability. Exploratory and summary statistics were obtained for all variables within the dataset. Data were examined for the presence of missing values, influential values and outliers, skew, and kurtosis. Continuous variables were described using mean and standard deviation, and categorical variables were described using frequency and percentage. *T*-test or ANOVA test was used to compare recovery scores of various socio-demographic characteristics, while Pearson product–moment correlations were used to analyze the correlation between clinical characteristics and recovery score. A multivariate linear regression was conducted to determine predictors of the total score of personal recovery with both socio-demographics (age, gender, marriage, education, occupation) and clinical characteristics (disability, functioning) included in the model. All data were analyzed using STATA version 16. Values of *p* < 0.05 were considered statistically significant (two-tailed test).

## Results

### Socio-Demographic Characteristics and Recovery

Among the 420 participants we approached, a total of 400 participants completed the questionnaires, with a response rate of 95%. Such a high response rate may be attributed to the research team of psychiatrists who have known the PLS well for a long time, which greatly increased study participation of PLS. Among the 20 non-responders, 6 refused the study, 10 were not able to communicate, 4 only completed the socio-demographic sheet. No significant differences in socio-demographics were found between those participants and non-participants. Demographic characteristics of the participants are summarized in Table 1. The sample had a mean age of 46.87 (SD: 10.99, range: 18–77), with the majority in the 46–59 age group (40.75%). Gender was equally distributed within the sample, with half men and half women. Although the majority (43%) were married, there were also a large portion of participants that were single (37.50%). The sample were generally well-educated, with over two thirds (67.75%) that had an education level of middle and high school, and 13.5% with a college degree or higher. Although well-educated, the unemployment rate among participants was high, 89.50%. A further comparison of recovery score among various demographic characteristics showed significant differences in all variables. Recovery score was higher in males than females (21.28 vs. 19.31, *p* = 0.035), and among those employed vs. unemployed (25.67 vs. 19.66, *p* < 0.001). Recovery was also higher among younger age and higher education participants, with the highest recovery score in the 18–35 age group (22.58) and college education group (24.93).

**Table 1:** Socio-demographic characteristics and association with recovery.

**Variables**		**Recovery score**		
**Socio-demographic characteristics**		***N* (%)**	**Mean ± Sd**	**P^b^**
Age	18–35	65 (16.25)	22.58 ± 9.74	
	36–45	121 (30.25)	22.34 ± 9.39	
	46–59	163 (40.75)	18.82 ± 8.67	
	60–100	51 (12.75)	17.24 ± 9.03	**<0.001**
Gender	Male	200 (50)	21.28 ± 0.67	
	Female	200 (50)	19.31 ± 0.64	**0.035**
Marriage	Single	150 (37.50)	21.68 ± 9.73	
	Married/cohabited	172 (43.00)	20.05 ± 8.85	
	Else^a^	78 (19.50)	18.15 ± 9.13	**0.022**
Education	Primary & below	75 (18.75)	17.29 ± 8.91	
	Middle & high	271 (67.75)	20.20 ± 9.05	
	College & above	54 (13.50)	24.93 ± 9.45	**<0.001**
Occupation	Unemployed	358 (89.50)	19.66 ± 0.49	
	Employed	42(10.50)	25.67 ± 1.33	**<0.001**

a *Else include divorced, separated, and widowed*.

b *t-tests were performed for analysis two group comparison, while ANOVA tests were performed for multiple group comparison; values in bold represents significant at p < 0.05 or p <0.01*.

### Clinical Characteristics and Recovery

Table 2 presents the clinical characteristics of the participants. Recovery score had a mean value of 20.29 (SD: 9.31, range: 8–40). Disability and functioning scores as measured by the WHODAS 2.0 and GAF, respectively, showed a mean score of 26.02 (SD: 10.22, range: 12–60) and 61.83 (SD: 13.58, range: 10–90). A further pairwise correlation between the three clinical indicators showed significant associations among all three variables. Recovery was significantly negatively associated with patient disability (*r* = −0.324, *p* < 0.001) and positively associated with functioning (*r* = 0.264, *p* < 0.001).

**Table 2:** Clinical characteristics and association with recovery.

**Variables**	**Mean ± Sd**	**RAS-8**	**WHODAS 2.0**	**GAF**
RAS-8	20.29 ± 9.31	1.00		
WHODAS 2.0	26.02 ± 10.22	−0.324^*^	1.00	
GAF	61.83 ± 13.58	0.264^*^	−0.397^*^	1.00

* *p < 0.001*.

*RAS-8, 8-item Recovery Assessment Scale; WHODAS 2.0, World Health Organization Disability Assessment Schedule 2.0; GAF, Global Assessment of Functioning*.

### Multivariate Predictors of Recovery

A multivariate linear regression was conducted to identify predictors of recovery. Among all 7 socio-demographic and clinical factors that were included in the model, five factors remained significant after controlling for all the other factors, including age, gender, education, patient disability and functioning (Table 3). In general, older age (*r* = −0.17, *p* < 0.001), being female (*r* = −2.29, *p* = 0.019), and higher disability (*r* = −0.22, *p* < 0.001) were independently associated with worse personal recovery, while having a college education (*r* = 5.49, *p* = 0.002), and higher functioning (*r* = 0.09, *p* = 0.017) were independently associated with better personal recovery. What is noteworthy is the strongest predicting effect of disability on recovery, with a standardized coefficient of −0.23. Every one-point increase in one's disability score decreased their recovery score by 0.22 (*B* = −0.22, 95% CI: −0.31, −0.12). Another interesting finding is the second strongest predicting effect of age on recovery, with a standardized coefficient of −0.20. Every one-point increase in age decreased their recovery score by 0.17 (*B* = −0.17, 95% CI: −0.26, −0.08).

**Table 3:** Multivariate linear regression of recovery.^a^.

**Variable**		**Coefficient**	**Standard coefficient**	**P**	**95% CI**
Age		−0.17	−0.20	**<0.001**	(−0.26, −0.08)
Gender	Male	ref			
	Female	−2.29	−0.12	**0.019**	(−4.20, −0.37)
Marriage	Single	ref			
	Married/cohabited	0.86	0.05	0.460	(−1.43, 3.15)
	Else	−0.81	−0.03	0.565	(−3.58, 1.96)
Education	Primary & below	ref			
	Middle & high	1.77	0.09	0.146	(−0.62, 4.16)
	College & above	5.49	0.19	**0.002**	(2.06, 8.91)
Occupation	Unemployed	ref			
	Employed	2.93	0.10	0.055	(−0.07, 5.93)
WHODAS 2.0		−0.22	−0.23	**<0.001**	(−0.31, −0.12)
GAF		0.09	0.13	**0.017**	(0.02,0.17)

a *Values in bold represents significant at p < 0.05 or p < 0.01*.

*RAS-8, 8-item Recovery Assessment Scale; WHODAS 2.0, World Health Organization Disability Assessment Schedule 2.0; GAF, Global Assessment of Functioning*.

## Discussion

This study sought to identify predictors of personal recovery among 400 PLS from 12 communities in Changsha City, Hunan Province, China. Our main findings were that participants had a relatively low personal recovery compared to that in other countries. Personal recovery was predicted by both socio-demographic and clinical characteristics. Younger age, being male, having a college education, lower disability, and higher functioning were all significantly and independently associated with better personal recovery.

The mean recovery score in this study was much lower than that of a similar community sample of PLS in Japan (41), and also lower than a summarized RAS score by a review on 77 studies using the RAS scale in various samples (42). This result may reflect that the concept of personal recovery is still in its early stage in Chinse culture and thus lack research and political attention. Also, it may reflect heterogeneity in recovery score among different samples, as shown in the current studies that recovery scores varies according to different socio-demographics. This finding brought public attention to the importance of personal recovery and indicates there is much room for improving personal recovery among PLS in China.

Initially the finding that younger age predicts better recovery appears to contradict previous research (25), but a closer look at the sample shows that with a mean age of 47 years and a mean illness duration of 21 years, individuals in our sample had already lived with schizophrenia for a long time. The finding that younger age was associated with better recovery may thus be reflective of the naturally debilitating course of schizophrenia. Another explanation may be that PLS represent a more heterogeneous group including not just a subgroup of participants that eventually will have a chronic illness course (mostly older PLS), but also a subgroup of participants that will recovery entirely (mostly younger PLS), as well as a subgroup of participants that will have a more intermittent illness course (mostly younger PLS). Considering age is a non-modifiable factor for personal recovery, it is strongly recommended that management of schizophrenia should focus on early detection, early treatment, and thus promote early recovery.

Our results also showed that males had better recovery scores than females, which is inconsistent with Ran et al.'s study of a Chinese cohort of PLS for 14 years which reported that males had poorer outcomes, including higher rates of mortality, suicide, and homelessness, as well as poorer family and social support, than females (43). One explanation for these differences may be the different conceptualization of recovery in our study that focused more on subjective feelings such as hope and optimism, instead of objective indicators in Ran at al's study. It is likely that although males have less favorable objective indictors of outcomes, they still personally feel better subjective personal recovery. Another possible reason may be the underreporting of poor outcomes by males due to masculine values in the culture (44). Males tend to report better outcomes due to gender roles where they were assumed to be strong and showing no weakness (45). As a result, men may report much better personal recovery than was actually the case. This novel finding has implications for further research to focus on gender-based recovery study and explore the mechanism behind the association of gender and recovery.

Consistent with the literature, our study showed that higher education predicts better personal recovery. One possible explanation is that education reflects premorbid functioning, such that those with higher education were those with better premorbid functioning, and thus enjoy better long-term outcomes and better personal recovery (46). Since education is generally not a modifiable factor after the onset of the disorder (24), more attention is needed for those PLS with lower education who may benefit from interventions that target other modifiable predictors. Another implication is that certain training may be provided to PLS with lower education level during the stable conditions to improve their literacy and recovery as well.

Previous studies have persistently suggested that employment is a strong predictor of personal recovery for PLS (24, 47). In the present study, we found significantly higher recovery scores among those employed than those not employed; however, these were only significant in the univariate analysis, with only trend effects observed for employment in the multivariate regression (*p* = 0.055). This may be explained by the high correlation between education and employment that reduced the predictive range of employment in the analyses. The effect of employment on recovery suggests occupational training as a facilitator for recovery and provides guidance for further development of rehabilitation programs that focus on occupational training and opportunities to improve recovery among PLS (48, 49).

Also consistent with the literature, functioning was found to be predictive of personal recovery (11, 50). One explanation may be that functioning and personal recovery represent two mutually exclusive, but complementary aspects of recovery (51). An alternate interpretation is that functioning and personal recovery have common determinants that results in co-variation of the two different variables (27). For instance, prior evidence has shown that several social factors influenced both patient functioning and personal recovery in the same way, and these social factors include social influence, self-esteem, sense of control, belonging, companionship, purpose and meaning, and perceived support availability (52). This finding provides implications for future studies to explore the association between functioning and recovery, as well as guide for intervention programs to focus on improving functioning to promote personal recovery.

Disability of the PLS had the strongest predictive effect on recovery. On the one hand, more disability represents more symptoms, which was associated with poorer prognosis and thus worse personal recovery (25, 28). On the other hand, more disability also reflects lower functioning, which as discussed above, predicts worse personal recovery. One of the implications of the findings is that personal recovery can be improved by reducing disability or providing supports to minimize its impact. This might include interventions that range from clinical (cognition, mobility, self-care) to social (getting along with people, life activities, and participation in society (33).

Finally, the study findings should be interpreted with caution due to a number of limitations. One concern is the accuracy of diagnosis for schizophrenia that was based on the CCMD-3 or the ICD-10 set by clinicians as part of general clinical practice instead of structured diagnostic instruments. Future research may use more structured instruments such as SCID or MINI. Secondly, we used a short-form of 8-item Recovery Assessment Scale (RAS-8) to measure personal recovery, even though the short-form has good psychometric properties. Future study may consider using the full RAS to examine recovery more comprehensively. A third limitation of this study relates to the cross-sectional study design which may preclude making causal inferences recovery and its predictors. Future research may benefit from using a longitudinal design to establish causality. A fourth limitation is that we did not include all potential variables that may predict recovery, such as social support, coping, stigma, among others. Future research is needed to include a more extensive list of potential factors predictive of recovery so as to assess their relative importance.

In conclusion, our study found that personal recovery was predicted by younger age, being male, having a college education, lower disability and higher functioning. Future interventions to improve recovery among PLS may be best served by focusing on intervention to reduce or minimize disability impairments and improving functioning, with targeted interventions for older persons, females, and individuals with lower education.

## Data Availability Statement

The raw data supporting the conclusions of this article will be made available by the authors, without undue reservation.

## Ethics Statement

The studies involving human participants were reviewed and approved by the Institutional Review Committee of the Xiangya School of Public Health of Central South University (No.: XYGW-2019-029). The patients/participants provided their written informed consent to participate in this study.

## Author Contributions

YY, XX, and Y-jL contributed to the conception and design of the study and contributed to data interpretation. YY, XX, MY, X-pG, T-xL, and GC contributed to the research conduction. MY, X-pG, and T-xL contributed to data collection. YY and XX contributed to data analyses and drafted the article while MY, X-pG, T-xL, GC, and Y-jL critically appraised it and revised it. All authors approved the final version of manuscript for submission and publication and agreed to be accountable for all aspects of the work.

## Conflict of Interest

The authors declare that the research was conducted in the absence of any commercial or financial relationships that could be construed as a potential conflict of interest.
